# Phosphorylation Changes in Response to Kinase Inhibitor H89 in PKA-Null Cells

**DOI:** 10.1038/s41598-019-39116-2

**Published:** 2019-02-26

**Authors:** Kavee Limbutara, Andrew Kelleher, Chin-Rang Yang, Viswanathan Raghuram, Mark A. Knepper

**Affiliations:** Epithelial Systems Biology Laboratory, Systems Biology Center, National Heart, Lung, and Blood Institute, National Institutes of Health, Bethesda, Maryland USA

## Abstract

Protein phosphorylation, mediated by protein kinases, plays a crucial role in cellular regulation. One of the most important protein kinases is protein kinase A (PKA). N-[2-p-bromocinnamylamino-ethyl]-5-isoquinolinesulphonamide (H89) is often used as a “PKA specific inhibitor” to study the involvement of PKA in signaling pathways. However, evidence from cell-free experiments has suggested that H89 can also inhibit other protein kinases. In this study, previously generated PKA-null and PKA-intact mouse cell lines derived from mpkCCD cells were treated with H89 over a range of concentrations commonly used in the literature, followed by mass spectrometry-based phosphoproteomics to globally assess changes in phosphorylation. From a total of 14,139 phosphorylation sites quantified, we found that 571 and 263 phosphorylation sites with significant changes in abundance in PKA-intact and PKA-null cells, respectively. Analyses of sequence logos generated from significantly decreased phosphorylation sites in PKA-intact and PKA-null cells both revealed a preference for basic amino acids at position −3 and −2. Thus, H89 appears to inhibit basophilic kinases even in the absence of PKA. Likely H89 targets include basophilic protein kinases such as AKT, RSK, AMPK and ROCK. We conclude that, in intact cells, H89 can affect activities of protein kinases other than PKA, and therefore responses to H89 should not be regarded as sufficient evidence for PKA involvement in a signaling process.

## Introduction

Protein phosphorylation is one of the most important post-translational modifications. This reversible modification plays a crucial role in cellular signal transduction^1^. Regulation of protein phosphorylation is mainly controlled by various kinases, a class of enzymes that catalyze the transfer of the phosphoryl group from adenosine triphosphate (ATP) to the hydroxyl group of proteins. Among over 500 known kinases^2^, protein kinase A (PKA) is one of the most studied. PKA catalytic subunits are coded by two separate genes, *Prkaca* and *Prkacb*. Functions of PKA span a wide-range of biological processes, including cell proliferation, differentiation, cell cycle control, metabolism, and transport^3^. Knowledge of PKA kinase-substrate relationship is thus essential to fully understand cellular signaling in both health and disease.

To determine if PKA is involved in a given cellular process, many investigators have relied on the use of small molecule kinase inhibitors. One such inhibitor is N-[2-p-bromocinnamylamino-ethyl]-5-isoquinolinesulphonamide (H89). It is widely claimed to be a potent and specific PKA inhibitor and is thus commonly used. However, evidence using purified recombinant protein kinases *in vitro* suggests that H89 may not be specific for PKA. Based on such *in vitro* kinase assays, H89 was shown to inhibit at least 8 other protein kinases (MSK1, PKBα, SGK, RSK1, RSK2, ROCK2, AMPK, and CHK1) by more than 80% at 10 µM^4^. In fact, H89 caused even greater inhibition of MSK1, RSK1, and ROCK2 than of PKA. A remaining question is to what extent H89 inhibits other kinases in intact cells. The recent development of mouse collecting duct cell lines in which CRISPR-Cas9 was used to delete both PKA catalytic subunit genes (PKA-null cells) allows this question to be addressed^5^. Here, we used large-scale quantitative phosphoproteomics analysis to test the effects of H89 in PKA-null and PKA-intact cells. Our results show that H89 causes broad effects on the phosphoproteome of PKA-null cells establishing that H89 has actions that are not specific to PKA.

## Methods

### Cell culture

The study utilized immortalized mpkCCD cells^6^ in which *Prkaca* and *Prkacb* gene expression was previously deleted (“PKA-null” cells) by introducing mutations using CRISPR-Cas9^5^. “PKA-intact” cell lines did not have deletion of either gene. We used three different PKA-null lines and three different PKA-intact lines in separate biological replicates. For each biological replicate, we also included up to three technical replicates. Cells were initially maintained in complete medium, DMEM/F-12 containing 2% serum and other supplements (5 μg/mL insulin, 50 nM dexamethasone, 1 nM triiodothyronine, 10 ng/mL epidermal growth factor, 60 nM sodium selenite, 5 μg/mL transferrin; all from Sigma). Cells were seeded on permeable membrane supports (Transwell) and grown in complete medium containing 0.1 nM 1-desamino-8-d-arginine-vasopressin (dDAVP, basal side only) for 4 d. Then, the medium was changed to simple medium (DMEM/F12 with dexamethasone, sodium selenite, and transferrin and no serum) with 0.1 nM dDAVP and maintained for 3 d. Transepithelial resistance (>2 kΩ-cm^2^) was measured by EVOM (WPI) prior to cell harvest to assure confluence and polarization. Cells were treated with 0.1, 1 or 10 µM H89 in DMSO (DMSO final concentration, 0.1%) for 30 min added to both apical and basal media. Controls were in 0.1% DMSO for 30 min.

### Immunoblotting

Cells were lysed with Laemmli buffer (1.5% SDS, 10 mM Tris, pH 6.8, protease and phosphatase inhibitors). Samples were homogenized using a QIAshredder (Qiagen). Protein concentration was measured using the BCA assay method. Samples were added with 5× loading buffer (7.5% wt/vol SDS, 30% vol/vol glycerol, 200 mM DTT, 50 mM Tris, bromophenol blue, pH 6.8) and incubated at 65 °C for 10 min. The denatured samples were subjected to SDS/PAGE. The proteins were transferred to nitrocellulose membranes and probed with anti-PKA antibodies (CST #4782). Blocking buffer and infrared fluorescence-conjugated secondary antibodies were obtained from LI-COR. Fluorescence images were visualized by a LI-COR Odyssey System.

### Phosphoproteomics

#### Sample preparation for total- and phospho-proteomics

Cells were washed three times with ice-cold PBS and then lysed with 8 M urea buffer (8 M urea, 50 mM Tris-HCl, 75 mM NaCl, 1× Halt protease and phosphatase inhibitors) and scraped into Eppendorf tubes, followed by sonication to solubilize proteins. Protein concentrations were measured using Pierce™ BCA Protein Assay Kit. Protein lysates were reduced with 20 mM dithiothreitol for 1 hour at 25 °C, and then alkylated with 40 mM iodoacetamide for 1 hour at 25 °C in the dark. The samples were diluted eight-fold with 20 mM triethylammonium bicarbonate (TEAB) buffer (pH 8.5) to reduce the urea concentration prior to digestion with Trypsin/LysC (Promega) (1:20 wt/wt.) overnight at 37 °C. The peptides were desalted using hydrophilic-lipophilic-balanced (HLB) extraction cartridges (Oasis) and quantified using Pierce™ Quantitative Colorimetric Peptide Assay. For each replicate, equal amounts of each sample (160–400 μg of peptide) were labeled using TMT10Plex Mass Tag Labeling Kit (Thermo Scientific) following the manufacturer’s instructions. Samples were combined and, after taking an aliquot for total proteomics, phosphopeptides were sequentially enriched following Sequential Enrichment from Metal Oxide Affinity Chromatography protocol (SMOAC, Thermo Scientific). We then fractionated the samples (12 fractions) using high pH reverse phase chromatography (Agilent 1200 HPLC System). Samples were then vacuum-dried and stored at −80 °C until analysis.

The dried peptides were re-suspended with 0.1% formic acid in LC-MS grade water (J.T. Baker) before mass spectrometry analysis. Total and phosphopeptides were analyzed using a Dionex UltiMate 3000 nano LC system connected to an Orbitrap Fusion Lumos ETD mass spectrometer equipped with an EASY-Spray ion source (Thermo Fisher Scientific). Peptides were introduced into a peptide nanotrap at a flow rate of 5 μL/min. The trapped peptides were fractionated with a reversed-phase EASY-Spray PepMap column (C18, 75 μm × 50 cm) using a linear gradient of 4 to 32% acetonitrile in 0.1% formic acid (120 min at 0.3 μL/min).

#### Mass spectrometry data processing

Raw mass spectra were searched against the *Mus musculus* UniProtKB^7^ reference proteome (Proteome ID: UP000000589, downloaded 17 April 2018, plus contaminant database) using MaxQuant^8^ 1.6.2.3. Reporter ion MS3 with TMT10plex was specified as labeling type and carbamidomethyl (C) was configured as fixed modifications. Lot-specific TMT isotopic impurities correction factors were used as recommended in TMT product data sheets. Variable modifications included phospho (STY), oxidation (M), and acetyl (Protein-N-term). Site localization scores generally exceeded 0.95 (Supplementary Dataset S2). False discovery rate was controlled at 1%. Trypsin/P and LysC/P were set as digestion enzymes with up to 2 missed cleavage allowed. Other parameters were set as default. Corrected reporter ion intensities from MaxQuant’s output files, “Phospho (STY)Sites.txt” and “proteinGroups.txt”, were used in phosphoproteome and total proteome analyses, respectively. Reporter ion intensities were transformed into log base 2 and median normalized for each channel. Of the 30 samples, 4 displayed significantly lower overall reporter intensities and were therefore excluded from downstream analyses. Technical replicates were summarized by taking the median value. Effect of H89 on total proteome was determined by log_2_ ratio of H89-treated group over control for each biological replicate. All analyses were performed using Perseus^9^, Excel, and R software.

### Analysis of H89 effects

A linear model for PKA-intact and PKA-null cells which included both H89 concentration and replicate number as predictor variables was independently fitted to each phosphosite.

For each replicate i, after logarithmic transformation of the data, we fit the equation

1 $$y={\beta }_{H89}{X}_{H89}+{b}_{i}$$

where β_H89_ is the slope estimated as one value over all replicates, but b_i_ is the intercept estimated separately for each replicate to deal with batch effects. Thus, for each phosphosite, we estimate four parameters, β_H89_, b_1_, b_2_ and b_3_. y is normalized log_2_ reporter intensity, X_H89_ is log H89 concentration, and β_H89_ is the fitted coefficient of X_H89_, representing the effect size of H89. Because log_10_(0) is undefined, we arbitrarily set the value of X_H89_ in control group (no H89) to zero. P values for β_H89_ were derived from moderated t-test against zero, implemented in Linear Models for Microarray Data (LIMMA) R package^10^. A significant change in phosphorylation for a phosphosite was defined as |β_H89_| > 0.15 and P < 0.05.

The phosphosites that were affected by H89 in each cell type were then categorized as increased or decreased based on the sign of β_H89_. Centralized peptide sequences (±10 amino acids) in each category were used to generate sequence logos using an updated version of PhosphoLogo^11^ (filtering χ^2^ < 0.01). Phosphosites with site localization probability (MaxQuant) less than 0.75 were excluded. The list of all quantified phosphosites, whether changed or not, were used to create a position-specific background matrix of amino-acid frequencies.

To test whether effects of H89 treatment on protein phosphorylation are enriched for any protein annotation terms, each protein was annotated with HPRD PhosphoMotif, gene ontology, KEGG, Pfam, GSEA, Corum, InterPro, PRINTS, PROSITE, Reactome, and SMART terms using Perseus software. 2D annotation enrichment analysis^12^ was then performed on β_H89_ of PKA-null and PKA-intact cells. Statistical test for enrichment of each term is based on MANOVA test on rank of each β_H89_. P-values were adjusted with Benjamini-Hochberg method to control false discovery rate at 0.01.

## Results

We reviewed the literature to determine the concentrations that have been used to investigate the role of PKA in various cellular processes (Supplementary Dataset S1). The range of concentrations was 0.1 to 100 μM and the median value was 10 μM. This compares with the reported *in vitro* inhibition constant (K_i_) value between 0.048 and 0.135 μM^4,13^. Consequently, in this study, we tested H89 concentrations ranging from 0 to 10 μM.

**Figure 1:**
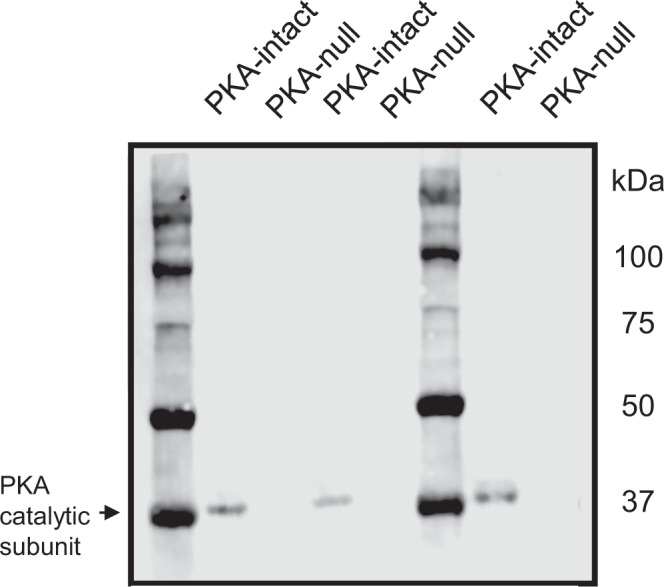
Immunoblot using a PKA antibody recognizing both PKA catalytic proteins shows absence of PKA catalytic subunits in PKA-null cells. The blot has been cropped and rotated for clarity. The full-length blot is presented in Supplementary Fig. S1.

Previously, the PKA-null cells were characterized by protein mass spectrometry and immunoblotting, showing that PKA-cα and PKA-cβ proteins were absent from the cells^5^. To confirm this finding in the same cells used in the present study, we carried out immunoblotting with an antibody that recognizes both PKA proteins. There was no detectable PKA band in any of the three PKA-null clones in contrast to the PKA-intact cells (Fig. 1).

Quantitative phosphoproteomics (TMT labeling) using the SMOAC phospho-enrichment protocol followed by LC MS/MS combined with high pH fractionation allowed quantification of 14,507 phosphosites including phosphosites in mono-phosphopeptides and combinations of sites in multi-phosphopeptides. This corresponds to 4,730 phosphoproteins. More than 90% of peptides identified were phosphopeptides after SMOAC. Pre-SMOAC enrichment samples were also analyzed, identifying 9,291 proteins.

**Figure 2:**
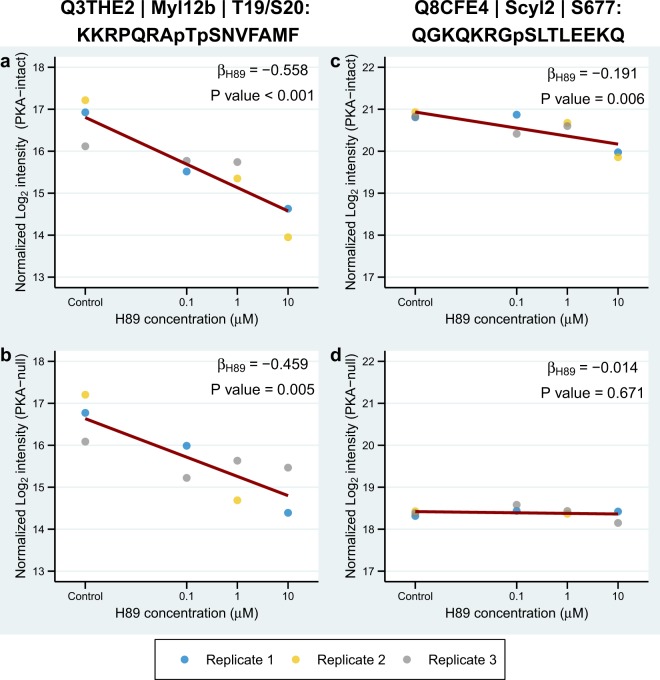
H89 effects on *Myl12b* and *Scyl2* phosphorylation. Examples of phosphosites from *Myl12b* and *Scyl2* are shown along with their associated linear models. Concentration of H89 (log_10_ scale) is plotted against log_2_-transformed intensity normalized for the batch effect. Dark red lines correspond to fitted linear models with slopes equal to β_H89_ and intercepts averaged from 3 replicates. UniProt accession numbers, gene symbols, phosphorylation positions, and centralized amino acid sequences are shown on the top. Phosphorylation at T19 and S20 in myosin regulatory light chain (*Myl12b*) are seen to be significantly decreased by H89 treatment in both PKA-intact (**a**) and PKA-null cells (**b**). Phosphorylation at S677 in SCY1-like protein 2 (*Scly2*) is inhibited by H89 in PKA-intact cells (**c**), but not in PKA-null cells (**d**), suggesting that this phosphosite is a PKA target.

To determine the effect of H89 on protein phosphorylation in each cell line, multiple linear regressions for phosphopeptide abundance versus H89 concentration and replicate number (to account for batch effect) were performed for each phosphosite (see Method). This effect was quantified as the coefficient (β_H89_) representing the slope of the log_2_(peptide intensity) versus log_10_(H89 concentration) lines, and P values were obtained using a moderated t-test for β_H89_. Figure 2 shows two examples of the data obtained. The first example shows results for a well-known pair of sites, T19 and S20 in myosin regulatory light chain (*Myl12b*), that are physiologically important in the regulation of non-muscle myosin II-A and II-B^14^. The phosphorylation is seen to be inhibited by H89 in both PKA-intact and PKA-null cells. This finding is consistent with the view that H89 inhibits one or more kinases among myosin light chain kinase, rho kinase and Pak kinases that have been documented to phosphorylate these sites^15–17^. The second example is a phosphorylation site, S677 in SCY1-like protein 2 (*Scyl2*; a component of AP2-containing clathrin coated structures) which was inhibited by H89 only in the PKA-intact cells. Phosphorylation at this site was previously shown to increase in response to vasopressin^18^ and was decreased in PKA-null cells versus PKA-intact cells^5^, suggesting that it is a PKA target site. The reader can examine any particular phosphorylation site interactively at https://esbl.nhlbi.nih.gov/H89/phosphosite.

**Figure 3:**
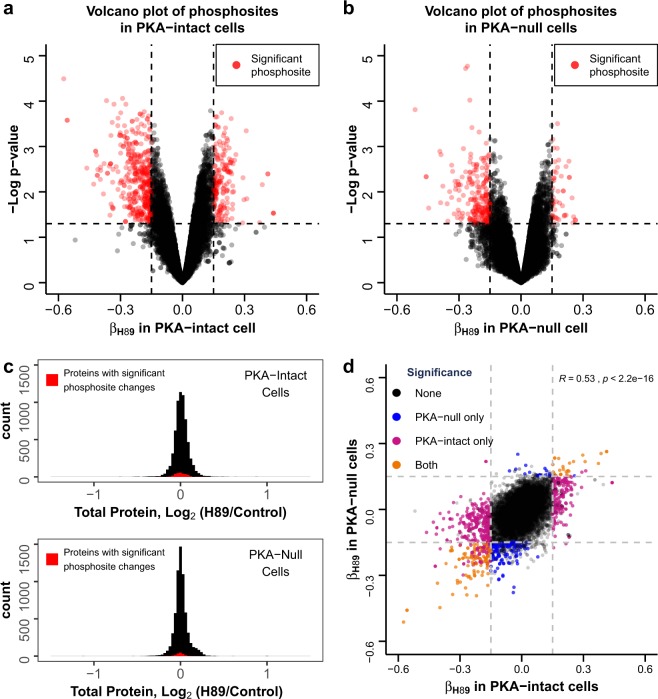
Effects of H89 on phosphoproteomes of PKA-intact and PKA-null mpkCCD cells. Volcano plots show global effects of H89 on phosphoproteome of PKA-intact cells (**a**) and PKA-null cells (**b**). Each point represents a phosphosite, x-axis denotes estimated effect size of H89 (β_H89_; positive and negative values mean H89 treatment increases and decreases phosphorylation, respectively). Y-axis represents negative of log_10_ P value for β_H89_. Significantly changed phosphosites, determined by P value < 0.05 and |β_H89_| > 0.15, are colored with red. (**c**) Distribution of total protein abundance ratios for PKA-intact and PKA-null cells show little or no change in response to H89 treatment. (**d**) Correlation between β_H89_ in PKA-intact and PKA-null cells is observed (Pearson’s correlation R = 0.53).

Overall, we identified 169 (1.2%) phosphosites that were significantly increased and 402 (2.8%) that were significantly decreased in response to H89 in PKA-intact cells (Fig. 3a). In PKA-null cells, 46 (0.3%) phosphosites were increased while 217 (1.5%) were decreased in response to H89 (Fig. 3b). Because of a short H89 treatment time used, these changes in phosphorylation are unlikely to be caused by changes in protein abundance. Indeed, Fig. 3c shows that, in general, total protein abundances did not change substantially. Plotting β_H89_ for PKA-intact versus PKA-null cells, a significant correlation was observed (Pearson’s correlation = 0.53, Fig. 3d). Notably, out of all phosphosites that underwent significant changes in the PKA-intact cells, none changed in the opposite direction in the PKA-null cells, and vice versa. Based on these findings, it is evident that H89 can inhibit protein kinases other than PKA at concentrations commonly used in the literature. Phosphorylation sites with the largest negative β_H89_ values are presented in Table 1 (PKA-intact cells) and Table 2 (PKA-null cells). Note that several of these sites are physiologically important, particularly sites in MAP/microtubule affinity-regulating kinase 2 (MARK2, a protein kinase with a role in epithelial polarity) and myosin regulatory light chain (discussed above). Note that some, but not all sites are found in both Tables 1 and 2. Sites with large positive β_H89_ values for are listed in Supplementary Table S1 (PKA-intact cells) and Supplementary Table S2 (PKA-null cells). The complete dataset is provided in Supplementary Dataset S2 and S3. The reader can examine any particular phosphorylation site or groups of sites in PKA-intact and PKA-null cells interactively at https://esbl.nhlbi.nih.gov/H89/volcano.

**Table 1:** Phosphorylation sites with largest negative β_H89_ in PKA-intact cells.

Gene	UniProt Accession	Protein Name	Position	β_H89_ in PKA-intact	Centralized Sequence
Mark2	E9QMP6	MAP/microtubule affinity-regulating kinase 2	208	−0.57	TFGNKLDTFCGSPPY
Myl12b	Q3THE2	Myosin regulatory light chain 12B	19, 20	−0.56	KKRPQRATSNVFAMF KRPQRATSNVFAMFD
Dek	Q7TNV0	Protein DEK	255	−0.46	SEDEEKESEEEQPPK
Smap	Q9R0P4	Small acidic protein	145	−0.43	SESEKEESAEELHAA
Ptpn13	G5E8B1	Tyrosine-protein phosphatase non-receptor type 13	340, 343	−0.42	RKKEGRYSDGSIALD EGRYSDGSIALDIFG
Htatsf1	Q8BGC0	HIV Tat-specific factor 1 homolog	613	−0.42	RVLDEEGSEREFEED
Msh6	P54276	DNA mismatch repair protein Msh6	200, 216	−0.41	LAVCDEPSEPEEEEE EVHEAYLSDKSEEDN
Ahnak	E9Q616	Desmoyokin	5590	−0.40	HGKLKFGTFGGLGSK
Tjp1	P39447	Tight junction protein ZO-1	277, 280, 284	−0.40	NVPDLSDSIHSANAS DLSDSIHSANASERD SIHSANASERDDISE
Leo1	Q5XJE5	RNA polymerase-associated protein LEO1	189	−0.40	DEEKMQNTDDEDRAQ

**Table 2:** Phosphorylation sites with largest negative β_H89_ in PKA-null cells.

Gene	UniProt Accession	Protein Name	Position	β_H89_ in PKA-null	Centralized Sequence
Mark2	E9QMP6	MAP/microtubule affinity-regulating kinase 2	208	−0.51	TFGNKLDTFCGSPPY
Myl12b	Q3THE2	Myosin regulatory light chain 12B	19, 20	−0.46	KKRPQRATSNVFAMF KRPQRATSNVFAMFD
Ahnak	E9Q616	Desmoyokin	5590	−0.45	HGKLKFGTFGGLGSK
Pak2	Q8CIN4	p21-activated kinase 2	197	−0.39	TKSIYTRSVIDPIPA
Pak2	Q8CIN4	p21-activated kinase 2	55	−0.39	KPRNKIISIFSGTEK
Eif4b	Q8BGD9	Eukaryotic translation initiation factor 4B	422, 425	−0.39	RERSRTGSESSQTGA SRTGSESSQTGASAT
Fbxo42	Q6PDJ6	F-box only protein 42	488	−0.38	SLAPRRGSLPDQKDL
Cfl2	P45591	Cofilin-2	3	−0.36	_____MASGVTVNDE
Pdlim5	Q8CI51	PDZ and LIM domain protein 5	228	−0.35	AEGQRRGSQGDIKQQ
Tns1	A0A087WQS0	Tensin 1	928	−0.35	QSHPLTQSRSGYIPS

**Figure 4:**
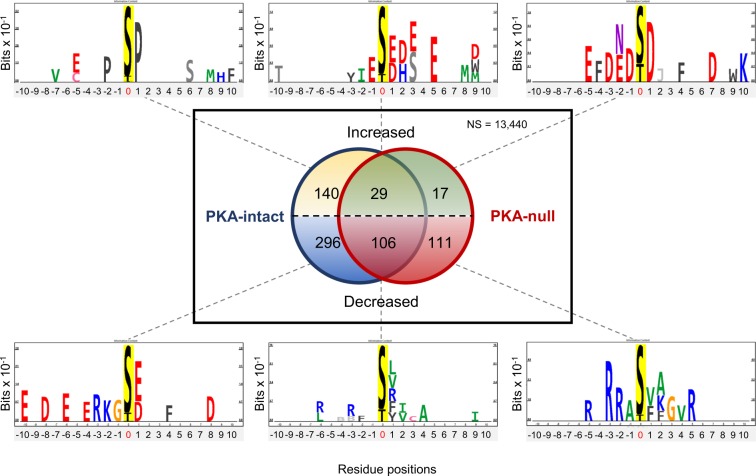
Sequence logos of phosphosites significantly changed by H89. Phosphosites are categorized according to significance and direction of changes. The Venn diagram shows both overlapping and non-overlapping significant phosphosites in PKA-intact (blue circle) and PKA-null (red circle) cells. The upper half represents increased phosphosites and lower half represent decreased phosphosites. Sequence logos, generated from significant phosphosites in each category, are shown on top and bottom with grey dashed lines connecting each sequence logo to the corresponding segment in Venn diagram). Phosphosites with site localization probability (MaxQuant) less than 0.75 were excluded from this analysis.

To further explore characteristics of H89 target phosphosites, we analyzed amino acid sequences surrounding each phosphosite using an updated version of PhosphoLogo^11^. Sequence logos based on information content for each subset of significant phosphosites were created as shown in Fig. 4. Decreased phosphosites in PKA-intact cells but not significantly changed in PKA-null cells showed typical basic amino acids (arginine [R] and lysine [K]) at position −2 and −3 (lower left). This motif is characteristic of basophilic kinases including PKA. There are also prominent acidic amino acids (aspartic acid [D] and glutamic acid [E]) suggesting that H89 may inhibit one or more acidophilic kinases e.g., casein kinases, indirectly downstream from PKA. Interestingly, in PKA-null cells, phosphosites decreased by H89 also showed prominent basic amino acids at position −2 and −3, consistent with inhibition of a one or more non-PKA basophilic kinases (lower right). Phosphosites that showed increases in phosphorylation in only PKA-intact cells showed a strong preference for proline at position +1, indicative of activation of MAP kinases or cyclin-dependent kinases (top left)^19^. Additional 2D annotation enrichment analyses (Supplementary Fig. S2) on various annotation terms showed a few terms with modest enrichment score. Notably, several basophilic kinase substrate motifs were also enriched in among decreased phosphosites in both PKA-null and PKA-intact cells, consistent with the conclusion that H89 inhibits primarily basophilic kinases.

**Figure 5:**
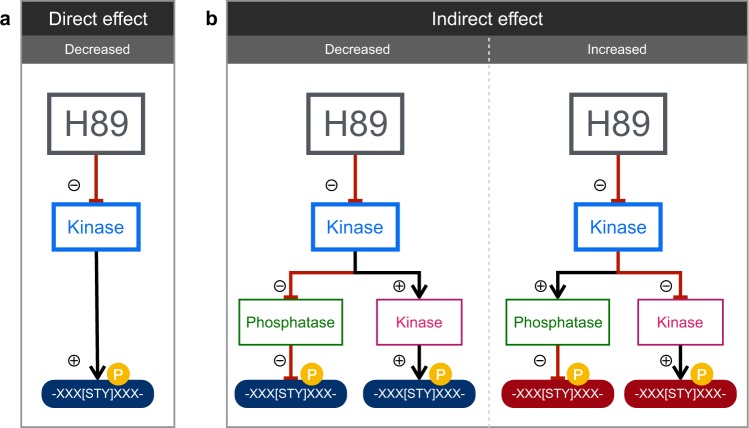
Simplified models for direct and indirect effects of H89 on phosphoproteome. (**a**) A direct effect of H89 inhibiting a kinase will result in an observed decrease in phosphorylation. (**b**) Alternatively, H89 can have indirect effects mediated via cascades of kinases and/or phosphatases. The affected phosphosites can be either increased or decreased, depending on downstream kinases and/or phosphatases. Plus sign indicates that phosphorylation activates the downstream kinase; minus sign indicates that phosphorylation inhibits the downstream kinase.

How can a kinase inhibitor increase phosphorylation at some sites? Increased phosphorylation presumably results from indirect effects on phosphorylation, e.g. in an inhibitory kinase cascade in which an upstream kinase (for example, PKA) is inhibited by H89, thereby resulting in activation of downstream kinases (Fig. 5). Additionally, some indirect effects of H89 could result from changes in phosphatase activities downstream from an inhibited kinase (Fig. 5).

What non-PKA protein kinases are inhibited by H89 to account for the extensive decreases in phosphorylation observed in PKA-null cells? Several types of information are pertinent to this question, namely the identification of kinases that are expressed in mpkCCD cells from prior proteomics^20^ and transcriptomics studies^5,21^, prior knowledge about kinase specificities compared to the sequence logos of inhibited sites shown in Fig. 4, and the prior data from cell-free kinase profiling^4,22,23^. Table 3 summarizes top ranking kinases inhibitable by H89 along with their associated prior information. These kinases can be considered as candidates for roles in phosphorylation of targets that have basophilic signatures and are inhibited by H89. Consistent with this view, phosphorylation of several known substrates of these kinases were significantly affected by H89 (Table 4). Additionally, analysis of total protein abundances in PKA-null versus PKA-intact cells (Supplementary Fig. S3) indicates that Maternal embryonic leucine zipper kinase (*Melk*) and Aurora-related kinase 2 (*Aurkb*) kinases are the basophilic kinases that are increased in abundance in the PKA knockout, providing a potential explanation for sites inhibited by H89 in PKA-null but not PKA-intact cells.

**Table 3:** H89 inhibitable kinases expressed in PKA-intact mpkCCD cells.

Gene Symbol	Kinase Family	Relative RNA Abundance^*^	Relative Protein Abundance^‡^	*In vitro* Percent Inhibition by H89 (10 µM)^†^
Prkacb	AGC	0.64	0.525	99
Rps6kb1	AGC	0.26	0.010	97
Akt1	AGC	3.53	0.122	95
Rps6ka5	AGC	0.22	0.028	95
Rock2	AGC	0.95	0.261	94
Pkn2	AGC	1.01	0.051	94
Rps6ka1	AGC	0.56	0.118	92
Akt2	AGC	0.58	0.024	88
Rps6ka3	AGC	0.17	0.170	81
Chek1	CAMK	0.44	0.004	76
Prkaa1	CAMK	0.64	0.779	74
Mark3	CAMK	1.98	0.061	71
Mink1	STE	0.42	0.001	66
Mark2	CAMK	0.74	0.195	65
Prkcz	AGC	0.24	0.005	65
Melk	CAMK	0.40	0§	62
Cdk2	CMGC	1.37	0.921	59
Aurkb	Other	0.72	0§	55
Map2k2	STE	1.38	0.591	54
Camk1	CAMK	0.69	0.000	53
Stk24	STE	1.09	0.507	51

^*^RNA abundance normalized by value for PKA catalytic α. From https://hpcwebapps.cit.nih.gov/ESBL/Database/PKA-KO/GeneSymbol.html.

^‡^Protein abundance normalized by value for PKA catalytic α. From https://hpcwebapps.cit.nih.gov/ESBL/Database/mpkCCD_Protein_Abundances/.

^†^Percent inhibition of purified enzyme. Data from http://www.kinase-screen.mrc.ac.uk/screening-compounds/341068.

^§^Upregulated in PKA-null cells.

**Table 4:** Non-PKA kinases that may be inhibited by H89 and their known substrates whose phosphorylation is decreased by H89 in present dataset.

Kinase Family	Likely H89 Target Kinases	Known Substrates	Phosphosite	Centralized sequence
AGC	Rps6ka1, Rps6ka3, Rps6ka5, Rps6kb1	Eif4b	S422	RERSRTGSESSQTGA
	Rock2	Myl12b	S20	KRPQRATSNVFAMFD
		Ppp1r12a	T694	ARQTRRSTQGVTLTD
	Akt1, Akt2	Pdcd4	S457	RGRKRFVSEGDGGRL
		Camkk2	S511	RREERSLSAPGNLLT
		Tbc1d4	T649	QFRRRAHTFSHPPSS
		Tbc1d1	T590	AFRRRANTLSHFPVE
		Srsf2	S189	RSRSRSRSRSPPPVS
		Eif4b	S422	RERSRTGSESSQTGA
		Gripap1	S631	TQTGDSSSVSSFSYR
		Thrap3	S51	RLSSRSRSRSYSPAH
		Prpf40a	S879	SKKRRHKSDSPESDT
**CAMK**	Prkaa1	Tbc1d1	T590	AFRRRANTLSHFPVE
	Mark2, Mark3	Baiap2	S367	KTLPRSSSMAAGLER
	Camk1	Myl12b	S20	KRPQRATSNVFAMFD
	Chek1	Krt8	S24	PRAFSSRSFTSGPGA
		Proser2	S43	CSSSRSRSFTMDDES
	Melk	Asap1	S10	SSASRLSSFSSRDSL
**Other**	Aurkb	Myl12b	S20	KRPQRATSNVFAMFD
		Hsp90aa1	S263	PEIEDVGSDEEEEEK

## Discussion

Knowing protein kinase/substrate networks is crucial to the understanding of cellular signal transduction. Over the years, H89 has been used to infer a role for PKA in many processes. In this study, we utilized unbiased phosphoproteomics and showed that many protein phosphorylation sites were altered in abundance by H89 treatment, both in PKA-intact and PKA-null cells. Because the peptide hormone vasopressin is the main stimulus to PKA activation in mpkCCD cells, the studies were done in the presence of the vasopressin analog dDAVP.

Although other investigations have already shown possible non-PKA inhibiting effects of H89, they were performed in cell-free systems. While these *in vitro* approaches are useful to study direct effects of small molecule inhibitors like H89 on each specific kinase, they may not predict the effects of H89 in living cells for a variety of reasons. The mechanism of H89-mediated kinase inhibition is believed to be through competition with ATP^4^. Therefore, differences in ATP concentration locally within the cell would modify H89 effects. In addition, the complex environment of the cell with multiple interacting proteins may modify responses to H89. To analyze the specificity of H89 toward PKA within cells, we took advantage of PKA-null mpkCCD cells previously generated in our lab using CRISPR-Cas9^5^ and their parent PKA-intact cells to investigate the effects of H89 on the phosphoproteome. Indeed, H89 in a concentration range of 0.1 to 10 μM had clear cut effects even in the PKA-null cells. Thus, care should be taken in formulating conclusions about the role of PKA in cellular processes based on H89 experiments.

What protein kinases are inhibited beyond PKA? The *in vitro* data points to MSK1, PKBα, SGK, RSK1, RSK2, ROCK2, AMPK, and CHK1^4,24^. The sequence logos generated from the current studies suggest as well that H89 may inhibit one or more basophilic kinases, which include AKT, RSK, AMPK ROCK, CHK, MARK, MELK, and AURB. As expected in *in vivo* studies, some of the effects of H89 on phosphorylation were likely indirect (Fig. 5). For example, in PKA-null cells, the phosphorylation of ribosomal protein S6 kinase alpha-1 (*Rps6ka1*), also known as RSK1, was decreased at an activating site, viz. S380 [AGAHQLFRGFpSFVATGLMEDD], which likely accounts for secondary decreases in phosphorylation of other proteins. This may include a Eukaryotic translation initiation factor 4B (*Eif4b*) double phosphopetide (S422 and S425), a known target of RSK1^25^, whose abundance was found to decrease.

One limitation of our approach is that kinase inhibition activity of H89 can only be detected if that kinase is both present and active in the cells. For that reason, it is likely that some phosphorylation sites that would be targeted by H89-sensitive kinases in other cell types, but which are unexpressed in mpkCCD cells, were unchanged. Yang *et al*. previously reported that less than 50% of all possible protein kinases were detected in mpkCCD cells using deep proteomic profiling^20^. Thus, in other cell types, H89 may inhibit a different set of kinases and consequently have distinct effects on the phosphoproteome.

In conclusion, we showed that H89 not only inhibits PKA, but also affects many protein phosphorylation events independent of PKA. Hence, a change in phosphorylation in response to H89 cannot alone be considered as sufficient evidence for a role for PKA.

## Supplementary information

Supplementary figures and tables

Supplementary dataset S1

Supplementary dataset S2

Supplementary dataset S3

## Data Availability

The mass spectrometry proteomics data have been deposited to the ProteomeXchange Consortium via the PRIDE partner repository with the dataset identifier PXD011167. (Username: reviewer38949@ebi.ac.uk, Password: LP0t3efh) Interactive data is accessible at https://esbl.nhlbi.nih.gov/H89/.
