# Supplementary material for: Phosphorylation Changes in Response to Kinase Inhibitor H89 in PKA-Null Cells
Source: Sci Rep. 2019 Feb 26;9:2814. doi: 10.1038/s41598-019-39116-2 (PMC6391403; doi:10.1038/s41598-019-39116-2)
Supplement: Supplementary file 1 — Supplementary figures and tables [file 41598_2019_39116_MOESM1_ESM.pdf]

# Supplementary Figures for Phosphorylation Changes in Response to Kinase Inhibitor H89 in PKA-Null Cells

Kavee Limbutara[&], Andrew Kelleher[&], Chin-Rang Yang[&], Viswanathan Raghuram and Mark A. Knepper[‡]

Epithelial Systems Biology Laboratory, Systems Biology Center,
National Heart, Lung, and Blood Institute,
National Institutes of Health, Bethesda, Maryland

[&]Contributed equally.

[‡]Correspondence to Mark A. Knepper, National Institutes of Health, Bldg. 10, Room 6N307, 10 CENTER DR, MSC-1603, Bethesda, MD 20892-1603,

Phone: (301) 496-3064, Fax: (301) 402-1443,

E-mail: knepperm@nhlbi.nih.gov

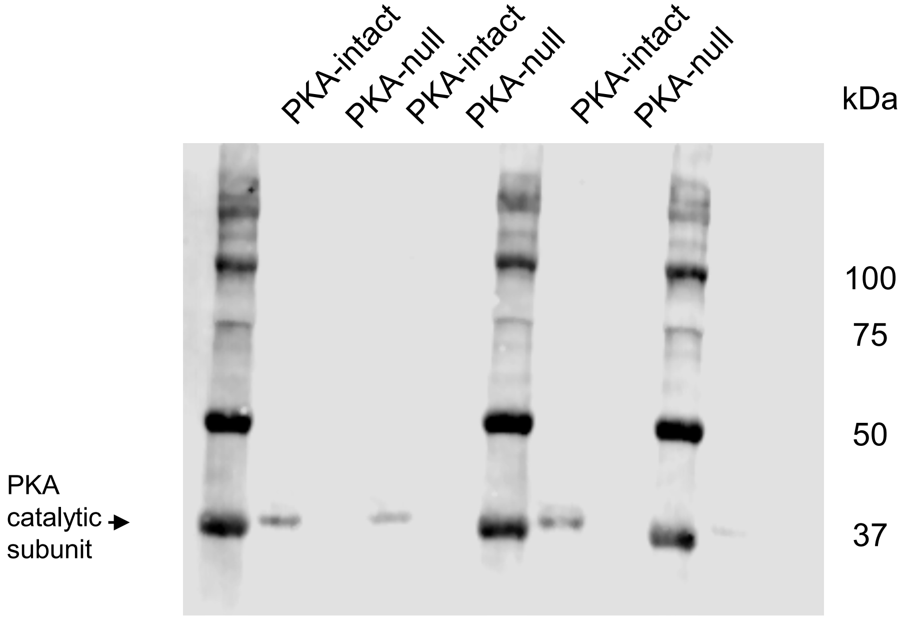


**Supplementary Figure S1.** Immunoblot using a PKA antibody recognizing both PKA catalytic proteins shows absence of PKA catalytic subunits in PKA-null cells.

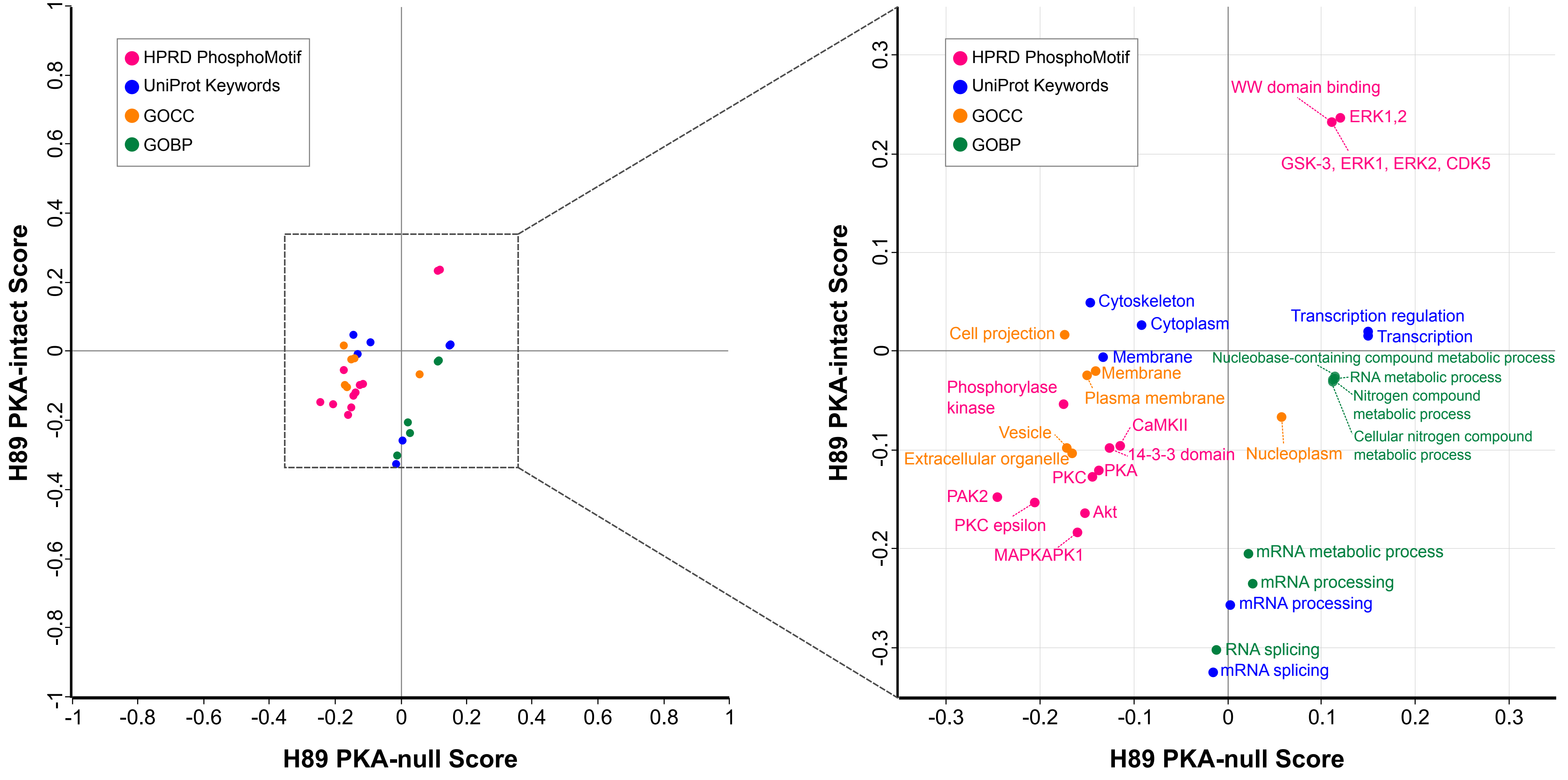


**Supplementary Figure S2. 2D annotation enrichment analysis.**

To compare effect H89 on phosphoproteome of PKA-intact and PKA-null cells, each phosphoprotein was annotated with HPRD PhosphoMotif, gene ontology, KEGG, Pfam, GSEA, Corum, InterPro, PRINTS, PROSITE, Reactome, and SMART terms. 2D annotation enrichment analysis was performed using Perseus software. Terms with adjusted p-value < 0.01 are shown in the scatter plot. X and y-axes correspond to enrichment score which calculated from rank of effect size of H89 on phosphosites (range from -1 to 1). All significant terms are clustered around the origin, suggesting minimal enrichment in any category. Consistent with sequence logos generated from significantly changed phosphosites, several non-PKA basophilic kinase motifs were enriched with negative score (decrease by H89) and proline-directed kinase motifs were enriched with positive score (increase by H89) in both cell types.

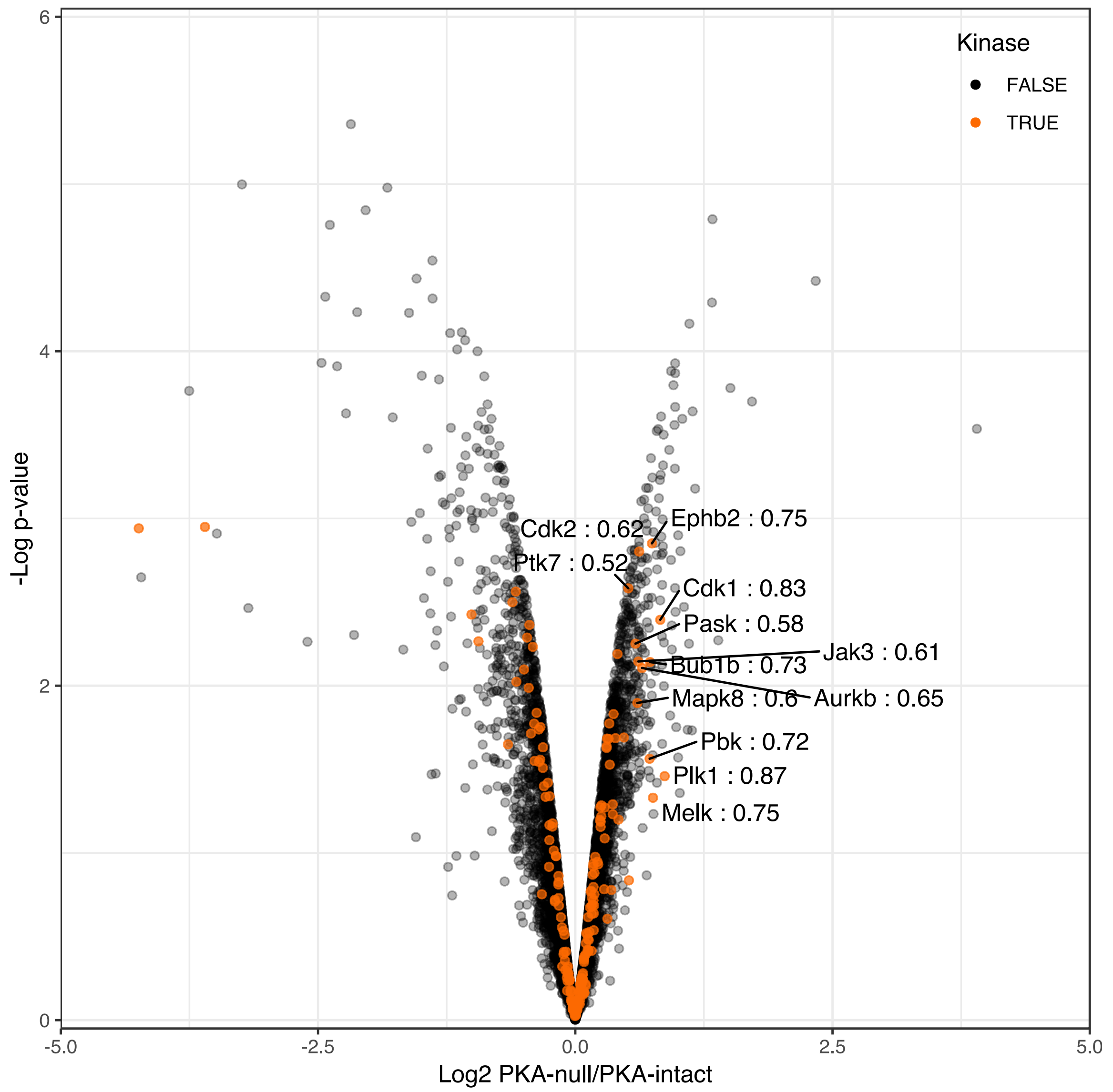


**Supplementary Figure S3. Up-regulated kinases in PKA-null cells.** A volcano plot shows log2 ratio of protein abundance (normalized log2 TMT reporter ion intensity) of PKA-null over PKA-intact cells (control group) on x-axis and negative of log10 p-value (moderated t-test, LIMMA) on y-axis. Kinases are colored in orange. Up-regulated kinases with p-value < 0.05 and log2 ratio > 0.5 are labeled with gene symbol and its log2 ratio value.

| Gene | Protein | Protein name | Position | $\beta_{H89}$ in PKA-intact | Centralized sequence |
|---|---|---|---|---|---|
| Snrnp200 | Q6P4T2 | U5 small nuclear ribonucleoprotein 200 kDa helicase | 2131, 2133, 2135 | 0.44 | VDVKEAETDSDSD__<br>VKEAETDSDSD____<br>EAETDSDSD______ |
| Chd2 | E9PZM4 | Chromodomain-helicase-DNA-binding protein 2 | 156, 165 | 0.41 | EKWKQDPSEDEQEQG<br>DEQEQGTSAESEAEQ |
| Iws1 | Q8C1D8 | Protein IWS1 homolog | 248, 263 | 0.39 | ELPKPRVSDSESEDP<br>QKGPASDSEAEDASR |
| Mcm3 | P25206 | DNA replication licensing factor MCM3 | 722 | 0.37 | PQVHTPKTDDSQEKT |
| Sept9 | Q80UG5 | Septin-9 | 85 | 0.31 | VDSLSQRSPKPSLRR |
| Llgl1 | A0A0R4J0S4 | Lethal(2) giant larvae protein homolog 1 | 988, 989 | 0.31 | ESCEGSPSSAHSKRA<br>SCEGSPSSAHSKRAD |
| Prr12 | E9PYL2 | Proline-rich protein 12 | 1555 | 0.30 | TAAVCGETDEEAGES |
| Mphosph10 | Q810V0 | U3 small nucleolar ribonucleoprotein protein MPP10 | 244 | 0.29 | DLFEDIDSDESEGGL |
| Hnrnpa1 | Q5EBP8 | Heterogeneous nuclear ribonucleoprotein A1 | 22 | 0.29 | KLFIGGLSFETTDES |
| Srsf7 | Q8BL97 | Serine/arginine-rich splicing factor 7 | 210 | 0.26 | ASLRRSRSGSIIGSR |

Supplementary Table S1 : Phosphorylation sites with largest positive $\beta_{H89}$ in PKA-intact cells.

| Gene | Protein | Protein name | Position | $\beta_{H89}$ in PKA-null | Centralized sequence |
|---|---|---|---|---|---|
| Chd2 | E9PZM4 | Chromodomain-helicase-DNA-binding protein 2 | 156, 165 | 0.26 | EKWKQDPSEDEQEQG<br>DEQEQGTSAESEAEQ |
| Iws1 | Q8C1D8 | Protein IWS1 homolog | 248, 263 | 0.25 | ELPKPRVSDSESEDP<br>QKGPASDSEAEDASR |
| Ptpn14 | Q62130 | Tyrosine-protein phosphatase non-receptor type 14 | 700, 705, 719 | 0.25 | PQYHHKKTFSDATML<br>KKTFSDATMLIHSSE<br>ESEEEEETLEAAPQV |
| Prr12 | E9PYL2 | Proline-rich protein 12 | 1555 | 0.25 | TAAVCGETDEEAGES |
| 8030462 N17Rik | Q0VAW6 | RIKEN cDNA 8030462N17 gene | 322, 323 | 0.23 | NEEINIASSDSEVEI<br>EEINIASSDSEVEIV |
| Utp18 | Q5SSI6 | U3 small nucleolar RNA-associated protein 18 homolog | 115, 118 | 0.22 | RGQLHGSSDESEVEN<br>LHGSSDESEVENEAK |
| Llgl1 | A0A0R4J0S4 | Lethal(2) giant larvae protein homolog 1 | 988, 989 | 0.22 | ESCEGSPSSAHSKRA<br>SCEGSPSSAHSKRAD |
| Mcm3 | P25206 | DNA replication licensing factor MCM3 | 722 | 0.21 | PQVHTPKTDDSQEKT |
| Wrnip1 | Q91XU0 | ATPase WRNIP1 | 91, 92 | 0.20 | ATPTAAESSEGEGEE<br>TPTAAESSEGEGEEG |
| Nucks1 | Q80XU3 | Nuclear ubiquitous casein and cyclin-dependent kinase substrate 1 | 144 | 0.20 | LMEDDDDSDYGSSKK |

Supplementary Table S2 : Phosphorylation sites with largest positive $\beta_{H89}$ in PKA-null cells.
